# Tobacco cessation, anti-tobacco education, and smoke-free schools: Findings from the Global Youth Tobacco Survey

**DOI:** 10.18332/tpc/193569

**Published:** 2024-11-21

**Authors:** Willie Rajvong, Yelena Tarasenko, Angela Ciobanu

**Affiliations:** 1 Department of Family and Community Medicine, Baylor College of Medicine, Houston, United States; 2 Faculty of Biostatistics, Epidemiology and Environmental Health Sciences, Jiann-Ping Hsu College of Public Health, Georgia Southern University, Statesboro, United States; 3 Division of Country Health Programs, World Health Organization Regional Office for Europe, Copenhagen, Denmark

**Keywords:** adolescents, tobacco cessation, GYTS, Global Youth Tobacco Survey, smoke-free schools, anti-tobacco education

## Abstract

**INTRODUCTION:**

Adolescents are especially vulnerable to the harmful effects of tobacco use. This study examined changes in tobacco use in schools, the provision of anti-tobacco education, and cessation efforts over time, and the importance of cessation support and education for cessation efforts among youth aged 13-15 years.

**METHODS:**

We performed secondary analyses of cross-sectional data from the latest two rounds of the Global Youth and Tobacco Survey (GYTS). Forty-five countries met the inclusion criteria for examining changes in quit attempts; 42 for receipt of cessation support; 28 for anti-tobacco education; 27 for tobacco use in schools, and 45 for the combined analysis of the association between cessation support and anti-tobacco education with quit attempts. To assess differences between the rounds, crude and adjusted prevalence estimates were compared as average adjusted predictions from univariate and multivariate logistic regressions. The association between quit attempts and other characteristics was examined using mixed effects binary logistic regression with a random intercept for the country.

**RESULTS:**

Percentages of youth who attempted to quit smoking (11/45), received cessation support (12/42), or saw others smoking on school premises (5/27) did not change in the majority of countries between survey rounds after adjusting for age and sex. Over half of the countries (15/27) reported significant changes in provision of anti-tobacco education between survey rounds, after adjustment. In 45 countries, adolescents who received help for quitting smoking (AOR=3.23; 95% CI: 3.02–3.45) or anti-tobacco education (AOR=1.13; 95% CI: 1.06–1.21) were more likely to attempt cessation than those without help or education (p<0.001).

**CONCLUSIONS:**

Despite the importance of cessation support and anti-tobacco education in promoting quit attempts among adolescents, many countries lack sufficient cessation initiatives for youth. Monitoring these indicators is necessary for guiding the development of public health interventions to reduce tobacco and nicotine product use among youths.

## INTRODUCTION

Tobacco is one of the world’s major preventable causes of morbidity and mortality^1^. Yet, in 2019, approximately 48.3 million or 12.1% of all adolescents aged 13–15 years globally reported having used any tobacco product^1^. Among all WHO regions, this ranges from 5.7 million or 8% of all adolescents in the Western Pacific region to 14.8 million or 13.6% of all adolescents in the South-East Asian region^1^. Quitting tobacco is an arduous process that often requires multiple attempts before success. According to the report by the United States (US) Surgeon General, for past year quit attempts of US high school students who smoked in 2017, 24.8% attempted to quit once, 19.5% attempted to quit twice, 18.2% attempted to quit 3–5 times, 10.5% attempted to quit 6–9 times, and 27% attempted to quit ≥10 times^2^. In a study based on the data from the Ontario Tobacco Survey, it took adults 30 attempts on average to successfully quit smoking for at least a year^3^.

While more recent studies on sources of smoking cessation for youth are urgently needed, existing research indicates smoking cessation can be reinforced with the help of friends and family, advice from health professionals, and cessation programs^4-6^. In a US study based on data from the 2012 National Youth Tobacco Survey, adolescents who had received parental advice had a significantly higher prevalence of quit attempts in the past year and intention to quit than those who had not received such advice^4^. A study of young adults in Canada found that physician advice to quit smoking, professional cessation resources such as self-help materials, behavioral therapies, pharmacotherapies, and participation in a local quit program had been associated with attempting to quit smoking^5^. In Taiwan, adolescents who observed anti-tobacco media messages, attended anti-smoking classes, or received cessation help, were significantly more likely to attempt to quit tobacco use than youth who did not^6^.

In a school environment, tobacco education programs can inform youth about the adverse effects of tobacco use and discourage them from initiating smoking^7-9^. Incorporating anti-tobacco education can fit into a school’s curriculum and provide an opportunity to reach most youths in a school’s area^7,8^. Many school-based tobacco education programs use a theoretical approach and are classified as having the following curricula: information-only, social competence, social influence, combined social competence/social influences, or multimodal^7^. Programs that use the information-only curriculum provide education on the misconceptions of tobacco and oppose beliefs that smoking is socially acceptable; those that use a social competence curriculum aim to reduce smoking experimentation by improving one’s social competence, and those that use a social influence curriculum teach adolescents to be aware and deal with social influences and peer pressure^7,8^. Adolescents in Zambia who received anti-tobacco education at school were less likely to have smoked in the past 30 days than those who did not^9^. A systematic review and meta-analysis of 50 randomized controlled trials of mostly US and European studies of school-based tobacco education programs found the combined social competence or social influences had been effective for reducing smoking initiation after any length of follow-up, and effective for the social competence curriculum after follow-up periods of more than 1 year^7^.

While several studies have assessed quit attempts among adolescents in different countries^3,4,10^, the current literature is lacking studies assessing global trends in quit attempts, help received for smoking cessation, anti-tobacco education in school, and seeing others smoke at school, used as a proxy for smoke-free schools. Additionally, prior studies have not reported on the association between quit attempts and help received for quitting smoking and received anti-tobacco education in school across different country settings. This study attempts to: 1) evaluate changes over time in tobacco cessation, assistance from personal or professional sources for quitting, provision of anti-tobacco education and exposure to smoking in schools; and 2) examine the association between quit attempts and received cessation support and anti-tobacco education in 45 countries, using the most recent GYTS data.

## METHODS

### Study design and data source

This study was based on secondary analyses of publicly available cross-sectional GYTS data. The analyses included: 1) a trend analysis of cessation efforts, anti-tobacco education, and smoke-free schools, using aggregated data from all respondents at the country level by survey round; and 2) a cross-sectional analysis of the association between quit attempts and help received for quitting smoking and anti-tobacco education using individual-level data from the most recently available GYTS. The latter included respondents from all countries listed in the Supplementary file Table 1, except Tajikistan and San Marino due to insufficient number of observations for the regression analyses restricted to youth who smoked. The GYTS is a global collaborative surveillance tool for monitoring tobacco use among youth and guiding implementation, and evaluation of tobacco control interventions in over 188 countries/sites across all six World Health Organization (WHO) regions^11^. The GYTS focuses on selecting the grades most associated with students aged 13–15 years. The surveys use a multi-stage clustered sampling design, with most countries using a two-stage sample design^11^. The overall response rates in countries included in the study are reported in Supplementary file Table 1. Youth demographic characteristics and information on receipt of anti-tobacco education in school, exposure to tobacco at schools, quit attempts, and help received for quitting smoking were extracted from the latest and previous rounds of the core GYTS in each country. Only responses from students aged 13–15 years were included in the analyses. Countries were included if :1) national data were available; 2) they had consistent data across years and indicators; 3) the latest round was conducted after 2015; and 4) rounds were no more than 7 years apart. While GYTS guidelines recommended conduct of surveys 5 years apart, we relaxed our inclusion criteria to allow for more countries and ensure that each WHO region was represented.

### Study variables

Quit attempts, help received for quitting smoking, anti-tobacco education, and seeing others smoke at school were operationalized using responses to core GYTS questions. For quit attempts and help received for quitting smoking, only students who currently smoked cigarettes (i.e. smoked at least 1 day during the past 30 days) were included in the analyses^1,9,12-14^. The number of observations per country in the combined analysis ranged from 41 in St. Vincent and the Grenadines to 6370 in Turkey, with the total n=27333. Tajikistan and San Marino had unweighted sample sizes of <35 current smokers and were excluded from the analysis for quit attempts and help received for quitting smoking, since prevalence estimates for these outcomes may be unreliable. The survey question: ‘During the past 12 months, did you ever try to stop smoking?’, was used to define quit attempts. Those who responded affirmatively were classified as having attempted^10,15^; all others were classified as no attempt. To ensure enough observations, those who responded affirmatively to the question: ‘Have you ever received help or advice to help you stop smoking’, were classified as having received help; all others as not having received help (Supplementary file Table 2). Responses (yes/no) to the question: ‘During the past 30 days, did you see anyone smoke inside the school building or outside on school property?’, were used to define having seen others smoke in school. Responses (yes/no) to question: ‘During the past 12 months, were you taught in any of your classes about the dangers of tobacco use?’, was used to define if participants received anti-tobacco education in school. For this question, approximately 10% of participants answered ‘I don't know’ across several countries; not all countries contained that answer choice. Those who responded ‘I don't know’ were removed from the analysis.

Sociodemographic characteristics, including age, sex, and pocket money, were included in the analyses as potential confounders *a priori*^6,16-19^. Country characteristics that were controlled for in the analyses, were WHO region^1^ and World Bank income classification^20^.

### Statistical analysis

To assess changes in prevalence of each outcome, separate analyses for each country were conducted. They were weighted to account for differences in the selection probability and to adjust for non-response bias for each country. In total, data from 47 countries were used. Data on quit attempts were available from 45 countries, on help received for quitting smoking from 42 countries, on anti-tobacco education from 28 countries, and on seeing someone smoke on school premises from 27 countries. Descriptive statistics included frequencies and percentages, and corresponding 95% confidence intervals (CIs). Age and sex adjusted prevalence was estimated as average adjusted predictions using margins command following the multivariate logistic regression. The difference in prevalence estimates between each country’s GYTS rounds were tested using a z-test.

To assess the association between students’ quit attempts and cessation support and anti-tobacco education, unweighted analyses based on the latest round of combined data from 45 countries were conducted. Mixed effects binary logistic regressions with random intercepts for each country were used. First, quit attempts were regressed on each independent variable at a time as fixed effects and random intercepts for each country. Then, three multi-level models were tested starting with Model 1 with all main independent variables as fixed effects, Model 2 with addition of age, sex, and pocket money, and Model 3 with addition of WHO region and World Bank Group income classification. Adjusted odds ratios (AORs) and 95% CIs were estimated.

All tests were two-tailed. Statistical significance was assessed at p<0.05. All analyses were conducted with Stata version 17.

The GYTS data are de-identified and publicly available; hence, their analysis does not constitute human subjects’ research. The study was exempt from review by the research ethics boards at the investigators’ institutions.

## RESULTS

### Changes in quit attempts for those who smoke

Percentages of students who attempted to quit smoking significantly changed in 12 out of 45 countries (Table 1). The adjusted prevalence of quit attempts significantly decreased in Belarus, Lithuania, Montenegro, Morocco, and Myanmar. The smallest decrease of 8.89 percentage points (pps) was in Lithuania, from 46.45% (95% CI: 39.96–52.94) in 2014 to 38.56% (95% CI: 34.11–43.02) in 2018 (p=0.05). The greatest decrease of 23.45 pps was in Morocco, from 56.38% (95% CI: 29.02–83.74) in 2014 to 32.93% (95% CI: 20.21–45.64) in 2018 (p=0.033). In the latest GYTS round, significantly more students reported trying to quit smoking in Croatia, Czech Republic, Grenada, Slovakia, Turkey, and Ukraine than in the previous GYTS round. The smallest increase of 16.55 pps was in Turkey, from 25.06% (95% CI: 18.95–31.18) in 2012 to 41.61% (95% CI: 37.83–45.39) in 2017 (p<0.001). The highest increase of 37.46 pps was in Ukraine, from 13.18% (95% CI: 8.02–18.35) in 2011 to 50.64% (95% CI: 38.77–62.51) in 2017 (p<0.001).

**Table 1:** Trends in quit attempts among students, aged 13–15 years, who currently smoke, in countries selected based on availability of the two latest rounds of GYTS data

*Countries (GYTS years)*	*Latest round*			*Previous round*					
	*Unweighted n (Yes/Total)*	*Unadjusted % (95% CI)*	*Adjusted % (95% CI) ^a^*	*Unweighted n (Yes/Total)*	*Unadjusted % (95% CI)*	*Adjusted % (95% CI) ^a^*	*p for unadjusted analysis*	*p for adjusted analysis ^a^*	*Changes occurred (in percentage points) ^d^*
Albania (2015–2020)	62/169	36.77 (28.69–44.85)	35.08 (26.96–43.20)	68/205	34.01 (27.79–40.42)	32.15 (25.74–38.55)	0.590	0.563	None
Argentina (2013–2018)	83/207	38.04 (24.26–51.81)	39.35 (27.74–50.96)	136/327	45.04 (37.65–52.44)	45.49 (37.92–53.05)	0.372	0.374	None
Azerbaijan (2011–2016)	28/57	48.69 (32.68–64.70)	52.04 (33.56–70.51)	12/55	23.04 (6.04–40.03)	29.28 (3.99–54.57)	0.033^b^	0.095	Increased (25.65; 22.76)
Belarus (2015–2021)	47/123	37.57 (28.49–46.65)	37.88 (29.54–46.21)	95/165	60.20 (49.90–70.51)	59.54 (49.28–69.79)	0.002^c^	0.002^c^	Decreased (22.63; 21.66)
Bhutan (2013–2019)	182/311	58.25 (52.39–64.10)	54.57 (44.00–64.93)	92/173	53.48 (47.96–59.00	49.49 (40.70–58.49)	0.239	0.244	None
Bolivia (2012–2018)	92/243	38.32 (30.28–46.36)	36.55 (27.03–46.06)	125/258	43.35 (25.07–61.62)	37.32 (24.38–50.25)	0.618	0.923	None
Brunei (2013–2019)	47/78	61.28 (49.84–72.71)	55.05 (41.12–68.98)	43/66	65.60 (52.29–78.91)	59.94 (41.32–78.56)	0.624	0.561	None
Croatia (2011–2016)	215/415	48.21 (43.17–53.26)	48.04 (42.75–53.33)	285/866	30.73 (27.74–33.72)	30.32 (27.17–33.47)	<0.001^b^	<0.001^b^	Increased (17.48; 17.72)
Czech Republic (2011–2016)	254/494	51.40 (46.46–56.33)	51.01 (45.85–56.17)	250/899	24.21 (19.92–28.50)	24.06 (19.66–28.47)	<0.001^b^	<0.001^b^	Increased (27.19; 26.95)
Fiji (2009–2016)	112/191	58.49 (52.58–64.40)	58.18 (52.27–64.09)	80/124	62.82 (54.36–71.28)	63.00 (55.51–70.50)	0.397	0.319	None
Georgia (2014–2017)	28/72	37.68 (25.19–50.16)	36.11 (23.28–48.93)	23/63	36.64 (28.58–44.50)	33.75 (24.57–42.93)	0.876	0.725	None
Grenada (2009–2016)	32/82	38.04 (26.83–49.25)	39.02 (27.51–50.53)	19/133	14.13 (7.87–20.38)	14.01 (7.35–20.66)	< 0.001^b^	< 0.001^b^	Increased (23.91; 25.01)
Guam (2014–2017)	59/104	57.03 (45.78–68.28)	59.83 (46.84–72.83)	64/96	69.79 (59.34–80.24)	69.03 (58.78–79.28)	0.103	0.238	None
Indonesia (2014–2019)	578/878	65.84 (61.91–69.76)	58.49 (52.02–64.96	459/677	65.96 (61.64–70.28)	58.29 (51.68–64.90)	0.966	0.950	None
Iraq (2014–2019)	64/138	44.00 (34.54–53.47)	44.39 (35.77–53.01)	23/68	34.64 (26.89–42.38)	34.86 (25.71–44.02)	0.128	0.113	None
Italy (2014–2018)	165/312	52.52 (46.32–58.72	52.43 (45.23–59.63)	153/312	49.77 (43.95–55.59)	49.90 (43.42–56.38)	0.521	0.545	None
Kyrgyzstan (2014–2019)	44/111	37.45 (26.80–48.10)	37.35 (26.20–48.50)	43/102	49.87 (39.20–60.54)	50.77 (36.23–65.32)	0.105	0.091	None
Laos (2011–2016)	108/230	45.31 (37.89–52.73)	49.72 (41.77–57.66)	174/304	57.48 (42.54–72.41)	52.51 (37.59–67.43)	0.364	0.372	None
Latvia (2014–2019)	267/512	55.23 (50.06–60.40)	54.87 (49.82–59.93)	311/610	51.72 (44.12–59.32)	51.29 (43.65–58.93)	0.448	0.438	None
Lithuania (2014–2018)	161/414	38.69 (34.16–43.22)	38.56 (34.11–43.02)	249/539	46.47 (39.98–52.96)	46.45 (39.96–52.94)	0.054	0.050^c^	Decreased (7.78; 7.89)
Marshall Islands (2009–2016)	75/153	44.70 (33.89–55.51)	47.03 (34.22–59.84)	27/50	54.19 (37.36–71.03)	58.38 (36.05–80.71)	0.350	0.268	None
Mongolia (2014–2019)	104/161	65.73 (56.99–74.47)	63.78 (52.43–75.14)	177/286	55.96 (46.93–64.98)	54.38 (44.02–64.74)	0.125	0.139	None
Montenegro (2014–2018)	79/246	31.43 (23.65–39.21)	32.93 (20.21–45.64)	110/261	60.24 (32.27–88.20)	56.38 (29.02–83.74)	0.052	0.033^c^	Decreased (28.81; 23.45)
Morocco (2010–2016)	16/52	18.63 (6.25–31.01)	16.52 (6.01– 27.03)	30/57	52.71 (38.89–66.53)	48.41 (33.29–63.53)	0.001^c^	0.002^c^	Decreased (34.08; 31.89)
Myanmar (2011–2016)	94/223	37.70 (29.91–45.49)	36.33 (24.71–47.95)	55/105	53.13 (43.95–62.30)	54.73 (41.14–68.31)	0.013^c^	0.001^c^	Decreased (15.43; 18.40)
Nicaragua (2014–2019)	263/569	45.82 (40.47–51.16)	46.02 (40.68–51.36)	149/322	46.04 (38.52–53.56)	45.68 (38.17–53.20)	0.962	0.943	None
Panama (2012–2017)	26/77	32.30 (21.91–44.09)	32.61 (21.80–43.41)	86/197	44.39 (36.71–52.08)	38.53 (29.61–47.45)	0.096	0.409	None
Paraguay (2014–2019)	32/100	29.00 (20.66–37.35)	28.91 (16.49–41.33)	91/225	41.73 (30.93–52.53)	41.11 (23.99–58.24)	0.067	0.098	None
Peru (2014–2019)	65/128	50.77 (42.40–59.15)	51.22 (42.53–59.90)	78/195	40.38 (32.25–48.50)	40.44 (32.64–48.24)	0.079	0.069	None
Philippines (2015–2019)	332/568	59.37 (55.08–63.67)	58.88 (54.51–63.24)	362/596	59.39 (54.02–64.77)	58.89 (53.70–64.07)	0.996	0.998	None
Qatar (2013–2018)	41/103	38.28 (27.73–48.84)	35.88 (27.79–43.97)	70/154	45.51 (38.91–52.11)	42.93 (33.51–52.36)	0.244	0.245	None
Republic of Moldova (2013–2019)	178/336	49.64 (41.66–57.62)	45.40 (37.17–53.63)	126/241	52.77 (43.98–61.55)	50.39 (42.07–58.72)	0.600	0.389	None
Romania (2013–2017)	188/361	51.99 (45.72–58.25)	47.98 (40.37–55.60)	161/297	54.15 (47.93–60.36)	51.83 (44.89–58.78)	0.628	0.384	None
Senegal (2013–2020)	21/72	28.44 (16.83–40.06)	31.03 (15.64–46.32)	10/37.0	21.80 (11.11–32.49)	16.97 (0.00–34.60)	0.399	0.153	None
Slovakia (2011–2016)	293/550	52.62 (47.20–58.05)	51.95 (46.78–57.12)	179/893	20.69 (18.11–23.27)	20.46 (17.81–23.12)	< 0.001^b^	< 0.001^b^	Increased (31.93; 31.49)
Slovenia (2011–2017)	38/120	27.77 (18.80–36.73)	28.53 (19.93–37.13)	89/265	30.84 (23.75–37.92)	31.54 (23.86–39.23)	0.590	0.605	None
St. Lucia (2011–2017)	28/68	42.17 (27.94–56.39)	41.19 (27.32–55.06)	23/70	33.48 (14.73–52.24)	31.67 (15.03–48.31)	0.464	0.376	None
St. Vincent and the Grenadines (2011–2018)	12/40.0	31.27 (17.10–45.44)	31.35 (16.61–46.09	54/125	44.11 (34.39–53.82)	47.53 (37.18–57.88)	0.141	0.086	None
Timor–Leste	107/243	44.20 (36.62–51.77)	42.22 (33.31–51.13)	181/445	40.81 (31.81–49.80)	38.29 (30.77–45.81)	0.560	0.488	None
Togo (2013–2019)	21/65	28.69 (13.17–44.20)	27.36 (12.44–42.27)	36/128	27.49 (17.28–37.70)	27.00 (15.53–38.47)	0.897	0.968	None
Trinidad and Tobago (2011–2017)	54/125	45.08 (36.14–54.02)	43.23 (34.39–52.08)	78/160	53.02 (42.27–63.78)	50.66 (39.52–61.80)	0.263	0.282	None
Tunisia (2010–2017)	52/122	43.35 (33.95–52.76)	38.02 (26.61–49.44)	33/81	41.14 (30.29–52.00)	36.66 (24.31–49.01)	0.759	0.843	None
Turkey (2012–2017)	3024/6315	46.15 (43.53–48.77)	41.61 (37.83–45.39)	122/424	26.28 (19.12–33.45)	25.06 (18.95–31.18)	<0.001^b^	<0.001^b^	Increased (19.87; 16.55)
Uganda (2011–2018)	27/68	58.27 (43.95–72.59)	53.30 (35.73–70.87)	32/75	45.17 (31.15–59.19)	45.34 (29.94–60.75)	0.195	0.395	None
Ukraine (2011–2017)	123/233	52.56 (41.06–64.06)	50.64 (38.77–62.51)	69/487	14.32 (9.63–19.00)	13.18 (8.02–18.35)	<0.001^b^	<0.001^b^	Increased (38.24; 37.46)

a Adjusted for age and sex.

b Significantly increased between rounds without the Bonferroni correction, resulting in p=0.001.

c Significantly decreased between rounds without the Bonferroni correction, resulting in p=0.001.

d Absolute difference for countries that have a significant change in at least one analysis is reported in percentage points; first for the unadjusted prevalence estimates, and then for the adjusted ones.

### Changes in help received for those who smoke

Percentages of students who received cessation support changed significantly in 12 out of 42 countries (Table 2). The adjusted prevalence significantly decreased in Argentina, Belarus, Bolivia, Montenegro, Czech Republic, Indonesia, Qatar, Slovakia, Togo, and Ukraine. The smallest decrease of 6.10 pps was in Indonesia, from 85.78% (95% CI: 80.38–91.17) in 2014 to 79.68% (95% CI: 74.14–85.22) in 2019 (p=0.018). The greatest decrease of 24.94 pps was in Togo, from 77.99% (95% CI: 67.75–88.24) in 2013 to 53.05% (95% CI: 33.07–73.03) in 2019 (p=0.019). Increases in adjusted prevalence of cessation support were in Albania, from 56.01% (95% CI: 47.92–62.12) in 2015 to 69.22% (95% CI: 61.62–78.62) in 2020 (p=0.017) and Mongolia, from 51.25% (95% CI: 38.89–63.61) in 2014 to 82.88% (95% CI: 72.59–93.17) in 2019 (p<0.001).

**Table 2:** Trends in cessation support or help received for quitting smoking among students, aged 13–15 years, who currently smoke, in countries selected based on availability of the two latest rounds of GYTS data

*Countries (GYTS years)*	*Latest round*			*Previous round*					
	*Unweighted n (Yes/Total)*	*Unadjusted % (95% CI)*	*Adjusted % (95% CI) ^a^*	*Unweighted n (Yes/Total)*	*Unadjusted % (95% CI)*	*Adjusted % (95% CI) ^a^*	*p for unadjusted analysis*	*p for adjusted analysis ^a^*	*Changes occurred (in percentage points) ^d^*
Albania (2015–2020)	115/165	69.88 (63.68–76.09)	69.22 (61.62–76.82)	118/206	56.75 (48.11–65.38)	56.02 (47.92–64.12)	0.016^b^	0.017^b^	Increased (13.13; 13.20)
Argentina (2013–2018)	86/206	41.15 (31.77–50.53)	42.02 (34.09–49.95)	195/327	61.55 (54.82–68.28)	62.43 (55.72–69.13)	0.001^c^	<0.001^c^	Decreased (20.40; 20.41)
Azerbaijan (2011–2016)	37/58	63.28 (41.86–84.70)	56.52 (32.88–80.16)	24/58	40.24 (28.26–52.21)	35.15 (21.98–48.31)	0.064	0.083	None
Belarus (2015–2021)	55/123	44.18 (33.49–54.87)	45.31 (33.68–56.94)	102/162	66.02 (55.89–76.15)	66.13 (54.70–77.56)	0.005^c^	0.006^c^	Decreased (21.84; 20.82)
Bolivia (2012–2018)	110/244	42.52 (34.51–50.52)	40.26 (31.74–48.78)	189/256	64.81 (43.19–86.43)	60.75 (41.10–80.40)	0.058	0.041^c^	Decreased (22.29; 20.49)
Brunei (2013–2019)	62/78	79.09 (68.25–89.93)	75.23 (61.71–88.76)	55/66	79.17 (68.13–90.22)	75.61 (59.46–91.75)	0.992	0.965	None
Croatia (2011–2016)	197/456	46.46 (40.62–52.30)	46.03 (40.33–51.73)	412/860	49.29 (45.18–53.40)	48.71 (45.04–52.38)	0.431	0.430	None
Czech Republic (2011–2016)	179/492	36.62 (31.36–41.87)	36.93 (31.55–42.30)	363/879	44.45 (39.25–49.65)	44.74 (39.47–50.02)	0.038^c^	0.040^c^	Decreased (7.83; 7.81)
Georgia (2014–2017)	51/72	71.32 (61.76–80.88)	65.66 (53.44–77.89)	38/64	61.39 (49.47–73.31)	54.78 (40.89–68.67)	0.194	0.171	None
Grenada (2009–2016)	47/82	56.50 (43.69–69.32)	55.45 (42.39–68.51)	83/130	64.44 (56.09–72.79)	62.96 (54.66–71.25)	0.306	0.336	None
Guam (2014–2017)	57/106	56.01 (46.76–65.25)	57.56 (44.23–70.89)	55/97	57.82 (46.06–69.59)	58.53 (46.53–70.53)	0.811	0.905	None
Indonesia (2014–2019)	724/879	82.59 (79.53–85.65)	79.68 (74.14–85.22)	596/676	87.81 (84.64–90.98)	85.78 (80.38–91.17)	0.021^c^	0.018^c^	Decreased (5.22; 6.10)
Iraq (2014–2019)	100/136	74.29 (63.33–85.25)	72.23 (62.08–82.38)	51/67	76.58 (65.73–87.43)	75.17 (62.35–87.98)	0.764	0.702	None
Italy (2014–2018)	138/312	46.32 (38.00–54.64)	47.48 (37.85–57.10)	138/313	44.64 (38.65–50.63)	46.34 (39.39–53.28)	0.744	0.823	None
Kyrgyzstan (2014–2019)	71/111	63.44 (48.78–78.10)	67.00 (50.49–83.51)	59/105	65.45 (50.30–80.60)	69.21 (54.24–84.18)	0.849	0.827	None
Laos (2011–2016)	163/228	71.29 (66.23–76.34)	69.49 (62.43–76.55)	207/305	68.50 (56.77–80.23)	67.95 (55.24–80.66)	0.665	0.792	None
Latvia (2014–2019)	277/512	55.40 (50.27–60.54)	55.95 (50.88–61.01)	315/613	51.46 (45.31–57.61)	51.68 (45.52–57.84)	0.328	0.288	None
Lithuania (2014–2018)	216/416	52.29 (46.57–58.01)	52.31 (46.55–58.08	279/539	51.80 (46.03–57.58)	51.79 (45.98–57.60)	0.905	0.899	None
Mongolia (2014–2019)	86/102	84.77 (76.48–93.06)	82.88 (72.59–93.17)	160/291	53.73 (45.45–62.02)	51.25 (38.89–63.61)	<0.001^b^	<0.001^b^	Increased (31.04; 31.63)
Montenegro (2014–2018)	116/246	48.94 (39.45–58.43)	53.53 (41.24–65.82)	150/261	71.24 (50.07–92.40)	72.19 (53.50–90.88)	0.059	0.027^c^	Decreased (22.30; 18.66)
Morocco (2010–2016)	38/58	72.71 (61.86–83.56)	65.32 (45.79–84.86)	42/56	74.78 (62.95–86.61)	71.11 (59.45–82.77)	0.796	0.601	None
Myanmar (2011–2016)	149/225	65.11 (57.73–72.48)	58.74 (45.86–71.61)	73/105	68.83 (58.35–79.31)	63.23 (47.85–78.61)	0.562	0.522	None
Nicaragua (2014–2019)	393/565	69.31 (65.35–73.28)	68.51 (64.68–72.35)	234/333	70.59 (64.65–76.53)	69.61 (63.59–75.63)	0.724	0.758	None
Panama (2012–2017)	48/76	63.66 (51.32–75.99)	64.19 (51.58–76.81)	123/193	68.36 (61.24–75.48)	67.51 (57.32–77.69)	0.510	0.658	None
Paraguay (2014–2019)	40/95	40.96 (26.61–55.30)	38.39 (26.04–50.75)	97/224	48.80 (36.31–61.28)	46.27 (29.59–62.95)	0.412	0.415	None
Peru (2014–2019)	58/129	47.19 (36.50–57.89)	48.25 (37.56–58.95)	92/194	54.96 (37.96–71.96)	55.22 (38.68–71.76)	0.441	0.482	None
Philippines (2015–2019)	465/575	82.44 (77.38–87.50)	81.58 (75.91–87.25)	494/598	82.21 (77.49–86.93)	81.33 (76.10–86.57)	0.947	0.943	None
Qatar (2013–2018)	63/105	60.10 (48.35–71.86)	56.00 (42.56–69.44)	118/159	73.91 (67.52–80.30)	70.21 (63.79–76.63)	0.044^c^	0.060	Decreased (13.81; 14.21)
Republic of Moldova (2013–2019)	227/337	67.91 (60.73–75.09)	65.28 (56.55–74.02)	162/242	71.70 (63.37–80.04)	67.43 (58.87–75.98)	0.492	0.733	None
Romania (2013–2017)	191/368	51.86 (45.69–58.04)	51.40 (42.47–60.33	168/296	57.12 (50.47–63.76)	57.29 (49.92–64.67)	0.253	0.220	None
Senegal (2013–2020)	45/72	63.16 (51.50–74.82)	64.71 (50.30–79.12)	23/36	61.69 (38.71–84.68)	58.94 (35.95–81.92)	0.909	0.673	None
Slovakia (2011–2016)	289/546	52.85 (47.85–57.86)	53.32 (48.41–58.24)	528/884	60.15 (56.01–64.29)	60.66 (56.33–64.99)	0.028^c^	0.03^c^	Decreased (7.30; 7.34)
Slovenia (2011–2017)	32/118	27.20 (18.47–35.94)	26.54 (17.51–35.57)	106/265	37.23 (31.10–43.36)	36.32 (30.06–42.59)	0.065	0.065	None
St. Lucia (2011–2017)	40/68	61.16 (50.19–72.13)	59.77 (47.78–71.77)	43/69	60.47 (46.45–74.50)	56.54 (44.20–68.87)	0.939	0.703	None
St. Vincent and the Grenadines (2011–2018)	28/41	68.80 (56.05–81.54)	67.85 (55.15–80.54)	93/126	74.50 (66.62–82.39)	76.32 (67.86–84.78)	0.451	0.280	None
Timor–Leste	215/246	88.36 (83.33–93.39)	85.33 (78.62–92.05)	400/446	89.07 (86.11–92.03)	86.93 (83.70–92.05)	0.806	0.640	None
Togo (2013–2019)	39/65	52.41 (33.06–71.75)	53.05 (33.07–73.03)	98/127	77.11 (68.85–85.37)	77.99 (67.75–88.24)	0.022^c^	0.019^c^	Decreased (24.70; 24.94)
Trinidad and Tobago (2011–2017)	67/124	57.75 (45.45–70.05)	54.93 (41.83–68.02)	106/156	66.64 (58.51–74.78)	62.29 (51.76–72.82)	0.234	0.352	None
Tunisia (2010–2017)	91/123	74.54 (67.90–81.19)	74.39 (64.82–83.97)	56/81	69.27 (57.13–81.41)	68.64 (54.30–82.98)	0.449	0.410	None
Turkey (2012–2017)	2974/6370	46.44 (44.15–48.72)	43.52 (39.55–47.49)	217/428	51.57 (43.51–59.63)	49.75 (40.98–58.52)	0.229	0.144	None
Uganda (2011–2018)	54/68	87.60 (77.17–98.03)	79.13 (60.54–97.71)	54/75	69.14 (47.48–90.81)	57.84 (38.00–77.69)	0.129	0.081	None
Ukraine (2011–2017)	120/231	49.80 (43.02–56.58)	47.18 (40.57–53.79)	363/480	74.12 (65.60–82.64)	70.22 (61.38–79.06)	<0.001^c^	<0.001^c^	Decreased (24.22; 23.04)

a Adjusted for age and sex.

b Significantly increased between rounds without the Bonferroni correction, resulting in p=0.001.

c Significantly decreased between rounds without the Bonferroni correction, resulting in p=0.001.

d Absolute difference for countries that have a significant change in at least one analysis is reported in percentage points: first for the unadjusted prevalence estimates, and then for the adjusted ones.

### Changes in anti-tobacco education

Percentages of students who received anti-tobacco education changed significantly in 15 out of 28 countries (Table 3). The adjusted prevalence significantly decreased in Albania, Belarus, Indonesia, Italy, Paraguay, Philippines, and Romania. The smallest significant decrease of 4.24 pps was observed in Paraguay, from 82.47% (95% CI: 78.27–86.67) in 2014 to 78.23% (95% CI: 75.60–80.85) in 2019 (p=0.043). The greatest decrease of 18.30 pps was in Albania, from 81.50% (95% CI: 78.82–84.18) in 2015 to 63.20% (95% CI: 60.30–66.10) in 2020 (p<0.001). Significant increases were observed among students aged 13–15 years in Bhutan, Georgia, Iraq, Mongolia, Peru, Republic of Moldova, Timor-Leste, and Togo. Peru had the smallest significant increase of 5.83 pps, from 66.50% (95% CI: 62.63–70.38) in 2014 to 72.33% (95% CI: 68.37–76.28) in 2019 (p=0.032). Georgia had the highest increase of 22.61 pps, from 34.46% (95% CI: 27.90–41.03) in 2014 to 57.07% (95% CI: 51.57–62.58) in 2017 (p<0.001).

**Table 3:** Trends in anti-tobacco education in schools in the past year among students, aged 13–15 years, in countries selected based on availability of the two latest rounds of GYTS data

*Countries (GYTS years)*	*Latest round*			*Previous round*					
	*Unweighted n (Yes/Total)*	*Unadjusted % (95% CI)*	*Adjusted % (95% CI) ^a^*	*Unweighted n (Yes/Total)*	*Unadjusted % (95% CI)*	*Adjusted % (95% CI) ^a^*	*p for unadjusted analysis*	*p for adjusted analysis ^a^*	*Changes occurred (in percentage points) ^d^*
Albania (2015–2020)	2193/3429	63.61 (60.61–66.61)	63.20 (60.30–66.10)	2623/3220	81.68 (79.04–84.31)	81.50 (78.82–84.18)	<0.001^c^	<0.001^c^	Decreased (18.07; 18.20)
Argentina (2013–2018)	345/1073	30.11 (21.14–39.08)	29.74 (22.51–36.98)	376/1833	21.59 (16.87–26.30)	21.64 (16.64–26.65)	0.097	0.082	None
Belarus (2015–2021)	1722/2210	77.38 (73.88–80.89)	77.50 (73.98–81.02)	2046/2253	91.86 (89.78–93.94)	91.86 (89.80–93.93)	<0.001^c^	<0.001^c^	Decreased (14.48; 14.36)
Bhutan (2013–2019)	1636/2030	80.09 (77.42–82.77)	80.21 (77.24–83.18)	840/1124	74.45 (71.09–77.80)	74.52 (70.40–78.64)	0.011^b^	0.012^b^	Increased (5.54; 5.69)
Brunei (2013–2019)	1116/1543	72.13 (68.90–75.35)	69.34 (65.77–72.91)	627/907	69.06 (65.82–72.31)	65.89 (61.61–70.16)	0.187	0.121	None
Georgia (2014–2017)	474/836	57.35 (51.65–63.04)	57.07 (51.57–62.58)	299/868	34.34 (27.70–40.98)	34.46 (27.90–41.03)	<0.001^b^	<0.001^b^	Increased (23.01; 22.61)
Guam (2014–2017)	733/909	80.30 (77.10–83.50)	76.70 (70.89–82.50)	510/633	79.51 (75.76–83.26)	78.05 (73.64–82.46)	0.751	0.652	None
Indonesia (2014–2019)	3171/4280	73.71 (70.93–76.49)	73.64 (70.85–76.43)	2916/3371	78.66 (76.00–81.32)	78.81 (76.12–81.49)	0.012^c^	0.010^c^	Decreased (4.95; 5.17)
Iraq (2014–2019)	812/1353	58.75 (51.43–66.07)	61.70 (54.81–68.59)	506/970	50.44 (46.26–54.62)	53.57 (48.95–58.20)	0.053	0.040^b^	Increased (8.31; 8.13)
Italy (2014–2018)	865/1341	64.37 (59.74–69.00)	61.97 (58.11–65.83)	895/1271	69.66 (65.15–74.17)	70.98 (66.83–75.13)	0.106	0.002^c^	Decreased (5.29; 9.01)
Kyrgyzstan (2014–2019)	3709/4647	80.70 (77.71–83.69)	81.52 (78.53–84.50)	2386/2925	81.87 (77.19–86.55)	82.30 (77.72–86.88)	0.677	0.722	None
Latvia (2014–2019)	1908/2819	70.10 (66.35–73.86)	69.98 (66.18–73.79)	1969/2949	68.31 (65.02–71.59)	68.02 (64.75–71.29)	0.474	0.434	None
Lithuania (2014–2018)	1521/2091	72.05 (67.68–76.43	72.54 (68.22–76.86)	1889/2625	71.41 (67.09–75.73)	71.99 (67.71–76.28)	0.836	0.858	None
Mongolia (2014–2019)	1768/3080	57.31 (53.02–61.61)	57.28 (52.98–61.59)	2304/4763	47.28 (43.31–51.25)	47.35 (43.30–51.39)	0.001^b^	0.001^b^	Increased (10.03; 9.93)
Montenegro (2014–2018)	1944/3149	61.49 (58.38–64.60)	60.09 (56.23–63.95)	1865/3165	63.52 (53.90–73.13)	61.73 (51.93–71.54)	0.690	0.738	None
Nicaragua (2014–2019)	3425/4863	70.00 (67.32–72.68)	70.07 (67.26–72.88)	1810/2650	69.33 (65.18–73.48)	69.24 (64.99–73.50)	0.789	0.743	None
Panama (2012–2017)	1205/1787	67.77 (64.35–71.19)	67.93 (64.63–71.23)	2302/3364	68.47 (65.39–71.55)	68.46 (65.37–71.55)	0.762	0.818	None
Paraguay (2014–2019)	2448/3124	77.43 (74.69–80.17)	78.23 (75.60–80.85)	3975/4792	82.47 (78.27–86.67)	83.17 (79.01–87.33)	0.049^c^	0.043^c^	Decreased (5.04; 4.94)
Peru (2014–2019)	1635/2325	71.65 (67.76–75.53)	72.33 (68.37–76.28)	1330/2032	65.84 (62.08–69.60)	66.50 (62.63–70.38)	0.036^b^	0.032^b^	Increased (5.81; 5.83)
Philippines (2015–2019)	3785/5878	64.90 (62.80–67.00)	65.02 (62.99–67.05)	3994/5412	72.87 (68.53–77.22)	72.85 (68.61–77.10)	0.001^c^	0.001^c^	Decreased (7.93; 7.83)
Qatar (2013–2018)	647/1168	55.68 (50.18–61.17)	56.19 (50.76–61.63)	763/1455	52.31 (48.46–56.17)	52.63 (48.87–56.38)	0.313	0.287	None
Republic of Moldova (2013–2019)	3216/3822	84.88 (83.06–86.70)	84.84 (83.01–86.68)	2304/3166	76.22 (72.83–79.61)	76.36 (72.88–79.85)	<0.001^b^	<0.001^b^	Increased (8.66; 8.48)
Romania (2013–2017)	2499/3670	67.67 (64.19–71.16)	67.89 (64.04–71.75)	2062/2827	73.12 (69.52–76.71)	73.32 (69.59–77.06)	0.033^c^	0.035^c^	Decreased (5.45; 5.43)
San Marino (2014–2018)	355/473	75.55 (71.35–79.75)	67.52 (62.24–72.79)	347/502	69.70 (65.21–74.19)	61.64 (57.81–65.48)	0.062	0.060	None
Senegal (2013–2020)	589/1366	41.50 (33.97–49.02)	44.84 (36.67–53.01)	197/459	33.65 (17.32–49.98)	38.56 (21.29–55.83)	0.384	0.496	None
Tajikistan (2014–2019)	1914/2468	78.18 (72.63–83.74)	78.51 (72.70–84.32)	1679/2120	78.97 (75.03–82.91)	79.21 (74.95–83.48)	0.818	0.834	None
Timor–Leste	811/1373	58.95 (53.22–64.68)	61.70 (55.88–67.51)	664/1387	48.07 (40.41–55.73)	51.55 (45.01–58.08)	0.027^b^	0.028^b^	Increased (10.88; 10.15)
Togo (2013–2019)	1175/1654	70.78 (61.42–80.13)	71.06 (61.80–80.32)	1074/2025	55.67 (49.65–61.68)	55.76 (49.72–61.81)	0.009^b^	0.007^b^	Increased (15.11; 15.30)

a Adjusted for age and sex.

b Significantly increased between rounds without the Bonferroni correction, resulting in p=0.002.

c Significantly decreased between rounds without the Bonferroni correction, resulting in p=0.002.

d Absolute difference for countries that have a significant change in at least one analysis is reported in percentage points: first for the unadjusted prevalence estimates, and then for the adjusted ones.

### Changes in seeing others smoke at school

Percentages of students seeing others smoke on school premises significantly changed in 6 out of 27 countries (Table 4). The adjusted prevalence significantly decreased in Albania, Argentina, Indonesia, Italy, Latvia, and Philippines. The smallest decrease of 8.51 pps was in Latvia, from 52.61% (95% CI: 47.83–57.40) in 2014 to 44.10% (95% CI: 40.19–48.00) in 2019 (p=0.008). The largest decrease of 17.10 pps was in Albania, from 46.36% (95% CI: 40.64–52.08) in 2015 to 29.26% (95% CI: 26.49–32.03) in 2020 (p<0.001). Significant increases were not observed.

**Table 4:** Prevalence of those who saw others smoke in schools in the past 12 months among students aged 13–15 years and comparison over time in countries selected based on availability of the two latest rounds of GYTS Data

*Countries (GYTS years)*	*Latest round*			*Previous round*					
	*Unweighted n (Yes/Total)*	*Unadjusted % (95% CI)*	*Adjusted % (95% CI) ^a^*	*Unweighted n (Yes/Total)*	*Unadjusted % (95% CI)*	*Adjusted % (95% CI) ^a^*	*p for unadjusted analysis*	*p for adjusted analysis ^a^*	*Changes occurred (in percentage points) ^d^*
Albania (2015–2020)	1139/3922	28.87 (25.99–31.74)	29.26 (26.49–32.03)	1602/3421	46.00 (40.33–51.68)	46.36 (40.64–52.08)	<0.001^c^	<0.001^c^	Decreased (17.13; 17.10)
Argentina (2013–2018)	400/1240	32.25 (24.22–40.27)	32.49 (24.37–40.61)	907/2022	48.52 (42.95–54.09)	48.74 (43.01–54.47)	0.002^c^	0.002^c^	Decreased (16.27; 16.25)
Belarus (2015–2021)	768/2649	29.08 (25.99–32.17)	28.97 (25.88–32.07)	801/2402	35.00 (29.43–40.57)	34.76 (29.17–40.36)	0.068	0.074	None
Bhutan (2013–2019)	1263/2332	55.04 (50.45–59.63)	55.33 (50.62–60.04)	691/1356	51.29 (46.31–56.27)	52.28 (46.89–57.67)	0.270	0.363	None
Brunei (2013–2019)	638/1536	43.99 (39.53–48.45)	45.27 (40.43–50.12)	443/914	49.83 (40.69–58.98)	51.21 (42.00–60.43)	0.257	0.229	None
Georgia (2014–2017)	459/927	49.33 (41.99–56.68)	49.26 (42.00–56.52)	501/945	53.78 (47.91–59.64)	54.06 (48.05–60.07)	0.341	0.309	None
Guam (2014–2017)	584/1075	56.13 (51.83–60.42)	64.88 (60.45–69.30)	444/725	61.69 (57.56–65.82)	63.65 (59.96–67.34)	0.067	0.666	None
Indonesia (2014–2019)	2878/5101	55.99 (52.07–59.91)	55.91 (52.03–59.79)	3007/4310	68.98 (64.14–73.82)	69.36 (64.55–74.16)	<0.001^c^	<0.001^c^	Decreased (12.99; 13.45)
Iraq (2014–2019)	667/1639	37.10 (24.83–49.36)	38.46 (31.68–45.24)	420/1236	31.85 (21.16–42.55)	33.63 (27.26–40.00)	0.516	0.281	None
Kyrgyzstan (2014–2019)	1857/5356	32.86 (28.72–37.01)	33.72 (29.50–37.94)	1238/3390	31.86 (27.26–36.46)	32.53 (27.93–37.12)	0.748	0.701	None
Latvia (2014–2019)	1601/3868	43.98 (40.04–47.92)	44.10 (40.19–48.00)	1933/3964	52.44 (47.66–57.23)	52.61 (47.83–57.40)	0.008^c^	0.008^c^	Decreased (8.46; 8.51)
Lithuania (2014–2018)	1334/2502	52.67 (46.03–59.32)	53.34 (46.81–59.88)	1862/3073	60.60(54.20–67.01)	61.40 (55.25–67.56)	0.091	0.080	None
Mongolia (2014–2019)	1893/3582	52.92 (49.86–55.98)	52.91 (49.85–55.98)	3564/6087	56.36 (53.29–59.43)	56.48 (53.50–59.47)	0.118	0.101	None
Montenegro (2014–2018)	2331/3827	54.93 (50.79–59.08)	50.58 (47.02–54.13)	2601/3848	62.36 (51.78–72.95)	55.60 (47.05–64.15)	0.196	0.275	None
Nicaragua (2014–2019)	2011/5407	38.36 (35.06–41.66)	39.80 (36.48–43.12)	1045/2911	36.91 (32.81–41.00)	37.54 (33.32–41.77)	0.585	0.411	None
Panama (2012–2017)	589/2066	28.92 (26.57–31.26)	28.73 (26.40–31.06)	1126/4028	27.94 (26.09–29.80)	27.57 (25.55–29.59)	0.515	0.461	None
Paraguay (2014–2019)	942/3437	27.16 (23.91–30.41)	26.81 (23.39–30.23)	1386/5106	27.91 (23.33–32.49)	27.27 (22.23–32.30)	0.791	0.874	None
Peru (2014–2019)	562/2546	20.35 (18.21–22.50)	20.84 (18.60–23.08)	470/2278	19.87 (16.89–22.85)	20.19 (17.03–23.36)	0.793	0.728	None
Philippines (2015–2019)	4091/6543	62.74 (59.77–65.72)	63.00 (60.05–65.94)	4229/5745	72.51 (68.54–76.49)	72.48 (68.57 –76.38)	<0.001^c^	<0.001^c^	Decreased (9.67; 9.48)
Qatar (2013–2018)	467/1540	30.14 (23.86–36.42)	29.85 (25.30–34.41)	454/1644	27.35 (19.47–35.23)	27.11 (25.30–34.41)	0.574	0.436	None
Republic of Moldova (2013–2019)	2288/4282	52.59 (47.14–58.04)	52.60 (47.17–58.03)	2207/3522	57.72 (53.65–61.79)	57.70 (53.70–61.70)	0.136	0.135	None
Romania (2013–2017)	1952/4275	45.46 (42.36–48.57)	45.55 (42.02–49.08)	1656/3281	50.91 (46.45–55.36)	50.97 (46.44–55.49)	0.049^c^	0.054	Decreased (5.45; 5.42)
San Marino (2014–2018)	265/544	49.34 (42.69–56.00)	47.51 (40.65–54.37)	293/529	55.37 (48.87–61.86)	53.81 (47.28–60.34)	0.201	0.174	None
Senegal (2013–2020)	1094/2399	42.09 (37.69–46.49)	41.08 (36.44–45.71)	286/786	37.32 (24.32–50.31)	35.50 (22.04–48.96)	0.487	0.404	None
Tajikistan (2014–2019)	448/2959	14.46 (12.08–16.85)	14.22 (11.93–16.50)	374/2294	16.28 (13.22–19.33)	15.72 (12.38–19.05)	0.352	0.427	None
Timor–Leste	917/1605	57.39 (51.47–63.32)	56.32 (49.93–62.70)	1183/1873	62.80 (54.67–70.93)	61.26 (54.42–68.10)	0.281	0.308	None
Togo (2013–2019)	378/2191	14.52 (10.26–18.78)	14.68 (10.40–18.96)	355/2794	12.66 (9.51–15.81)	12.77 (9.52–16.02)	0.485	0.477	None

a Adjusted for age and sex.

b Significantly increased between rounds without the Bonferroni correction, resulting in p=0.002.

c Significantly decreased between rounds, resulting in a p=0.002.

d Absolute difference for countries that have a significant change in at least one analysis is reported in percentage points: first for the unadjusted prevalence estimates, and then for the adjusted ones.

### Combined analyses

In all analyses (Table 5), the GYTS survey respondents who had not received cessation support and anti-tobacco education were less likely to attempt quitting smoking than those respondents who did. Adjusting for the individual and country-level characteristics (Supplementary file Table 3), those who received help for quitting smoking had 3.23 times higher odds of attempting smoking cessation in the past year (95% CI: 3.02–3.45) than those who did not (p<0.001), and those who received anti-tobacco education had 1.13 times higher odds of attempting to quit smoking in the past year (95% CI: 1.06–1.21) than those who did not receive such education (p<0.001).

**Table 5:** Combined individual-level analysis of the relationship between quit attempts and help received for quitting smoking and anti-tobacco education in schools in study participants, aged 13–15 years, who currently smoke

*Charasteristics*	*OR (95% CI)*	*p*	*Model 1 AOR (95% CI)*	*p*	*Model 2 AOR (95% CI)*	*p*	*Model 3 AOR (95% CI)*	*p*
**Received help for quitting smoking**								
No ®	1		1		1		1	
Yes	3.39 (3.21–3.59)	<0.001	3.55 (3.33–3.78)	<0.001	3.44 (3.22–3.67)	<0.001	3.23 (3.02–3.45)	<0.001
**Received anti-tobacco education**								
No ®	1		1		1		1	
Yes	1.23 (1.16–1.31)	<0.001	1.12 (1.05–1.20)	0.001	1.12 (1.05–1.20)	0.001	1.13 (1.06–1.21)	<0.001
Age (years)								
13 ®	1				1		1	
14	1.26 (1.17–1.36)	<0.001			1.18 (1.08–1.29)	<0.001	1.12 (1.02–1.23)	0.015
15	1.64 (1.53–1.76)	<0.001			1.50 (1.38–1.64)	<0.001	1.44 (1.32–1.57)	<0.001
**Sex**								
Male ®	1				1		1	
Female	0.77 (0.73–0.81)	<0.001			0.81 (0.76–0.87)	<0.001	0.80 (0.75–0.86)	<0.001
**Pocket money**								
Other pocket money ®	1				1		1	
Most pocket money	1.14 (1.05–1.23)	0.002			1.13 (1.02–1.24)	0.016	1.11 (1.01–1.23)	0.030
**Region**								
Americas ®	1						1	
Europe	1.09 (0.77–1.54)	0.640					0.94 (0.69–1.26)	0.661
Southeast Asian	1.77 (1.07–2.94)	0.027					1.72 (1.08–2.76)	0.024
African	0.62 (0.34–1.13)	0.116					0.49 (0.20–1.24)	0.134
Eastern Mediterranean	0.94 (0.56–1.59)	0.812					0.84 (0.53–1.32)	0.457
Western Pacific	1.92 (1.22–3.00)	0.005					1.52 (1.04–2.19)	0.032
**Income**								
Low ®	1						1	
Lower middle	1.19 (0.54–2.64)	0.671					0.51 (0.19–1.43)	0.202
Upper middle	1.29 (0.59–2.84)	0.528					0.69 (0.24–2.02)	0.500
High	1.67 (0.75–3.69)	0.207					1.05 (0.36–3.07)	0.934

AOR: adjusted odds ratio. Model 1: received help for quitting smoking and anti-tobacco education. Model 2: plus age, sex, and pocket money. Model 3: plus WHO region and country-level income. ® Reference categories.

Female respondents had 0.80 times lower odds to attempt to quit smoking in the past year (95% CI: 0.75–0.86) than male adolescents (p<0.001). Study participants aged 14 years had 1.12 times higher odds (95% CI: 1.02–1.23) and those aged 15 years had 1.46 times higher odds (95% CI: 1.33–1.59) of quit attempts than adolescents aged 13 years (p=0.015 and p<0.001, respectively). Study participants with the most pocket money had 1.11 times higher odds of attempting to quit smoking in the past year (95% CI: 1.01–1.23) than adolescents with less pocket money (p=0.030). Finally, study participants from the Western Pacific region and the South-East Asian region, were significantly more likely to attempt to quit smoking in the past year than those from the Americas region (Table 5).

## DISCUSSION

We have found that the percentages of youth who had attempted to quit smoking, received support for quitting, or observed others smoking on school premises did not change significantly in most of the countries included in the study. There were significant changes in over half of the countries for youths who received anti-tobacco education; however, adjusting for multiple comparisons using the Bonferroni correction narrowed these results to just over onethird of the countries. Based on the most recent GYTS rounds in 45 countries, among adolescents aged 13–15 years, the adjusted prevalence of quit attempts ranged from 16.52% in Morocco to 63.78% in Mongolia, and the adjusted prevalence of help received for quitting smoking ranged from 26.54% in Slovenia to 85.33% in Timor-Leste. While the adjusted prevalence of quit attempts in adolescents who currently smoke has increased in 6 countries, this is not necessarily indicative of successful quit attempts. Not all quit attempts are successful; one study reported the median prevalence of relapse for adolescents who attempted to quit was 34% within 1 week, 56% within 1 month, 89% within 6 months, and 92% within one year^21^. On average, it takes multiple attempts before smoking cessation is successful^3,21^. Among common motivations for attempting to quit smoking are aid received for cessation from friends and family, or aid and cessation resources provided by a professional organization^4-6^.

The adjusted percentage of adolescents seeing others smoke inside school buildings or outside on school property ranged from 14.22% in Tajikistan to 67.80% in Italy. Secondhand smoke causes serious diseases and premature death among non-smokers^22^. Smoke-free laws in public places such as schools are protecting everyone’s right to breathe clean air^22^. Studies showed that smoke-free laws were associated with decreases in smoking prevalence and smoking initiation among youth^23,24^. Additionally, a systematic review found smoke-free policies had been consistently associated with reduced smoking behaviors, exposures to secondhand smoke, and adverse health outcomes^25^.

The adjusted percentage of adolescents who received anti-tobacco education ranged from 27.72% in Argentina to 84.84% in the Republic of Moldova, based on the most recent GYTS round conducted in 28 countries. Nearly an equal number of countries reported significant increases (8) and decreases (7) in the adjusted analysis. However, participants who received anti-tobacco education were more likely to attempt to quit smoking across all individual-level analyses. A systematic review found school-based programs with a strong theoretical foundation and incorporating formative research in their design, were effective in reducing smoking among adolescents^26^.

Lack of progress in quit attempts, provision of cessation support and anti-tobacco education is concerning, especially in light of our individual-level findings that GYTS survey respondents who received this support and education were significantly more likely to attempt to quit smoking in the past year than those who did not. Prior studies also found those who had received advice for quitting smoking or used professional cessation programs and resources were more likely to intend and attempt to quit smoking^4-6^. As the majority of studies on school-based tobacco education focus on preventing smoking initiation among non-smokers, a meta-analysis of 25 RCTs found school-based cessation programs and programs by trained teachers and educators, had significantly reduced smoking behaviors, including initiation and continuation^27^. A strong relationship between adolescents and their teachers and educators has been seen as a key factor in preventing juvenile delinquency and enhancing the adolescent’s capacity to manage and overcome challenging behaviors^27^. Re-emerging research on school-based interventions should consider the importance of positive effects of such close bonding, and how best to prepare teachers and educators to foster their students’ fight for a tobacco-free wellbeing. For example, WHO has developed a global guide and toolkit to create nicotine- and tobacco-free schools by providing engaging activities, communication materials, and practical examples from schools worldwide that have adopted such interventions^28^.

### Strengths and limitations

Our study has several strengths. First, the GYTS data collection is based on probability sampling, including random selection of classes. Hence, survey findings are generalizable to students aged 13–15 years in each country. Second, the questionnaire uses standardized methodology allowing for cross-country comparisons. This study has several limitations. First, it is susceptible to information bias, since the GYTS data are self-reported. Second, current smokers were limited to those who smoked in the past 30 days, and since quit attempts were assessed for the past 12 months, all quit attempts by those who smoke may not be fully captured. Third, we performed multiple comparisons of outcomes. With significance assessed at p<0.05, there is a risk of Type-1 error due to the number of statistical analyses performed. In the notes for Tables 1–4, we have reported the Bonferroni-corrected p-values. Using them as a benchmark would further narrow any changes reported as significant ones between the GYTS rounds.

## CONCLUSIONS

Cessation support and anti-tobacco education are positively associated with quit attempts of adolescents-GYTS respondents. However, there appears to be a lack of progress in terms of increasing percentage of youth attempting to quit smoking and receiving smoking cessation and anti-tobacco education. This lack of progress tends to correspond to limited up-to-date research on effective cessation interventions for youth, especially considering changing landscape of tobacco products over the last decade and differences of low versus high resource settings. Continuous monitoring of cessation-related indicators and all types of tobacco and nicotine products is necessary to help guide the development and implementation of public health interventions to curtail tobacco and nicotine product use among youth.

## Data Availability

Data sharing is not applicable to this article as no new data were created.
